# The impacts of earthquakes on air pollution and strategies for mitigation: a case study of Turkey

**DOI:** 10.1007/s11356-024-32592-8

**Published:** 2024-02-27

**Authors:** Alessandra Zanoletti, Elza Bontempi

**Affiliations:** INSTM and Chemistry for Technologies Laboratory, Department of Mechanical and Industrial Engineering, University of Brescia, Via Branze, 38, 25123 Brescia, Italy

**Keywords:** Earthquake, Air pollution, PM, Asbestos, Polychlorinated biphenyls, Chromium

## Abstract

This study delves into the repercussions of the 2023 earthquake in Turkey, particularity its impact on air pollution. A year post-event, it is evident that scientific literature has paid limited attention to monitoring the situation. However, the release of hazardous substances, such as asbestos, lead, and other toxins, from damaged structures poses a significant threat by contaminating nearby air, soil, and water sources, thereby jeopardizing ecosystems and public well-being. The improper disposal of waste post-earthquake and the presence of mining and oil refinery sites in the region contribute to potential air pollutants. These circumstances create challenging environments conducive to the spread of respiratory diseases, with potential long-term health and social consequences. Unfortunately, existing data gaps hinder a comprehensive understanding of the situation. This paper pioneers the reporting and analysis of data regarding potential sources of air pollution resulting from the earthquake in Turkey. It also pinpoints gaps in knowledge, outlining areas that demand further investigation. To effectively prevent and mitigate air pollution risks and associated health concerns linked to earthquakes, strategic recommendations are proposed. A key suggestion is the establishment of post-disaster air pollution monitoring systems capable of swiftly identifying emerging health issues, facilitating efficient responses, and curtailing potential long-term effects of the disaster. The paper underscores the necessity for continuous health monitoring of the affected population to mitigate possible adverse impacts on human health. These strategies play a pivotal role in reducing the likelihood of air pollution, supporting emergency response and recovery initiatives, and fostering new dedicated scientific studies.

## Introduction

Scientists indicate that the rise in global greenhouse gases within the atmosphere has played a role in escalating extreme weather occurrences, thereby fostering the circumstances conducive to the emergence of natural disasters and other unfavorable incidents (Prohaska and Peters 2019).

Natural disasters can result in substantial physical devastation, leading to loss of life, and significant environmental transformations. Certain disasters, like forest fires and volcanic eruptions, contribute to air pollution by releasing substantial amounts of gases and particles into the atmosphere, thereby escalating the risk of respiratory and heart diseases in the affected population. However, there is limited knowledge about the secondary consequences of air pollution induced by natural events such as earthquakes, floods, tsunamis, landslides, hurricanes, and the like. This lack of understanding stems from the fact that secondary air pollution is not a direct outcome of the disaster itself (Sekine and Shinohara 2017).

On February 6, 2023, a 7.8-magnitude earthquake hit Syria and Turkey, followed by multiple aftershocks and a subsequent 7.6-magnitude earthquake (Zilio and Ampuero 2023). Turkey is prone to natural disasters. The most frequent natural disasters that the nation experiences include earthquakes, landslides, floods, and avalanches. According to the data, a devastating earthquake strikes Turkey every 5 years, placing it fourth in the world for big earthquake frequency (AFAD 2018). The country gets a score of 5.0 on the INFORM index, making it a high-risk location with an upward trend in risk over the past few years (AFAD 2018). According to national statistics, 8274 meteorological disasters were recorded in the country between 2010 and 2021, with 2021 recording the highest numbers (Doğan et al. 2021).

**Fig. 1:**
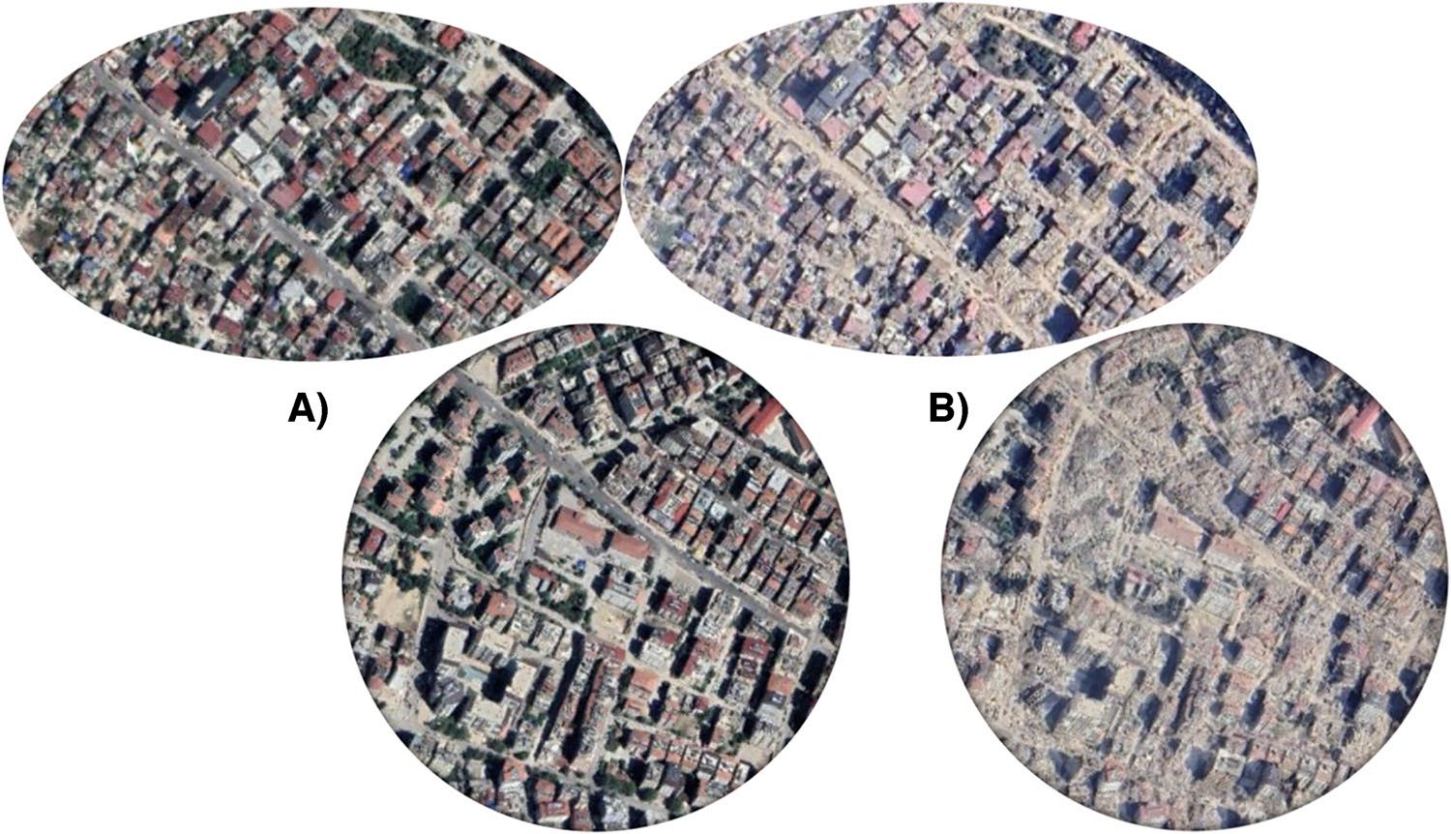
Satellite images of Antakya before (September 27, 2021, A) and after (February 15, 2023, B) the earthquake (courtesy of Maxar)

By comparing satellite images taken before and after the earthquake, the extent of devastation along the affected area becomes evident, as seen in Fig. 1. The earthquake generated an enormous amount of debris, surpassing the scale of other major disasters. The United Nations estimates that the calamity produced at least 10 times more rubble than the previous major earthquake in Turkey in 1999.

Several papers have reported on the environmental effects of earthquakes, highlighting the significant impact on the natural surroundings and human settlements (Khlef et al. 2022; Bilgili and Çetinkaya 2023; Martins et al. 2023; Gonzalez et al. 2023; Lin and Wang 2023; Mavroulis et al. 2023; Ozkula et al. 2023). These studies have examined various aspects such as the changes in landforms, soil liquefaction, landslides, and the disruption of ecosystems. Additionally, they have investigated the implications for public health, infrastructure, and environmental monitoring. However, little attention was devoted to air pollution originating from these natural disasters. Nevertheless, to the immediate devastation consequences, it is also crucial to underscore the potential secondary repercussions of such a disaster. In past occurrences, individuals affected by disasters or their communities have experienced health issues stemming from air pollution triggered by the disaster within the human living environment. For example, comparable catastrophic incidents involving building collapses have exposed substantial health risks associated with the generation of significant amounts of dust and powders.

The terrorist attacks on the World Trade Center in New York City on September 11, 2001, resulted in the complete collapse of the two WTC towers, which served as enormous point sources of gaseous and particulate air pollution (Manning 2001). The smoke contained volatile organic compounds and fine particles and aerosols, while the dust fraction contained parts of ceiling tiles, carpets, concrete, adhesives, asbestos, chromium, lead, titanium, and many other elements and materials (Manning 2001). However, whether there were unusually toxic ingredients in the plumes is largely unknown. Air pollution levels increased after 9/11 in Windsor, Ontario, as shown by higher levels of SO_2_ and CO concentration levels 1 month and 6 months post September 11, 2001, compared to the same period 1 year earlier and 1 year later (Luginaah et al. 2006). Some studies found an increased risk of pulmonary fibrosis and long-term cardiovascular disease in people with medium and very high levels of exposure to World Trade Center dust (as for example firefighters), which included metals (Li et al. 2019; Cohen et al. 2019). These studies were performed more than 15 years after the disaster. Disaster preparedness and response planning must be given top priority to support the healthcare system and people’s security. Moreover, great attention must be also devoted to preventing environmental pollution, which may affect the health of the population in the next years.

Low attention was devoted by the research community concerning the potential health risks associated with the Turkey and Syria earthquake.

This is one of the first works that delve into the environmental concerns arising from the 2023 earthquakes in Turkey, aiming to address the various challenges and provide valuable insights for future preparedness and response efforts. The paper places a particular emphasis on some critical aspects concerning air pollution: potential contaminants resulting from improper waste disposal practices following earthquakes and the potential impact of dust and powders originating from the existing Turkey mining sites. Unfortunately, specific works treating the particles released in the atmosphere as a consequence of a natural disaster are mainly addressed to volcano eruptions (Ruano-Ravina et al. 2023), which involves different contaminants.

In the aftermath of earthquakes, one of the pressing issues that emerge is the inadequate management of waste generated by the disaster. Improper disposal of debris and building materials can lead to the release of hazardous substances into the environment, posing risks to both human health and ecological systems. This study critically assesses the possible source of air contamination caused by post-earthquake waste disposal practices and explores strategies to ensure proper waste management, including appropriate sorting, storage, and disposal methods. The objective is to address the issue and provide recommendations to curb the dissemination of pollutants, thereby minimizing their adverse impacts on the environment, public health, and ecosystems (Núñez-Delgado et al. 2023).

For this aim, this study presents a range of strategies to effectively prevent and mitigate health issues associated with air pollution due to earthquakes. In this context, it is crucial to consider ecological rationality, which asserts that the rationality of a decision is contingent upon the circumstances in which it is made, aiming to achieve specific goals within a given context (Coccia 2020). By analyzing the possible environmental effects and proposing solutions to these multifaceted concerns, this study aims to contribute to the body of knowledge on earthquake secondary effects and provide valuable recommendations for policymakers, emergency responders, and stakeholders involved in disaster preparedness and response.

## Study design

To investigate the possible sources of air pollution after this extreme event requires careful consideration of the geographical characteristics of the area and the intensity of the disaster. Turkey, officially recognized as the Republic of Türkiye, is situated at the intersection of Southeast Europe and West Asia. It holds a distinctive geographic position, spanning across both Asia and Europe.

A 7.8-magnitude earthquake struck southeastern Turkey, near the Syrian border in 2023.

Since the devastating earthquake in 1999 that struck the densely populated eastern Marmara Sea region near Istanbul, resulting in thousands of fatalities, the 2023 earthquakes have been the deadliest to hit Turkey. As of February 12, according to the disaster and emergency management authorities of the country, over 33,000 people had lost their lives, and thousands more were injured in both Turkey and Syria as a consequence of the earthquakes that occurred in Turkey’s southeast on February 6 (Rasheed et al. 2023). Table 1 reports the number of collapsed buildings after this earthquake, by city. Eighteen thousand buildings collapsed after the two major earthquake events. Hatay experienced the most extensive devastation, with 5700 collapsed buildings. Subsequently, Kahramanmaraş and Gaziantep were affected, with nearly 3800 and 3400 buildings reduced to rubble, respectively (source: Ministry of Environment UACC 2023).

**Table 1:** Number of collapsed buildings after the Turkey earthquake in 2023, by city

City	Buildings
Adana	18
Adyaman	2349
Diyarbakr	175
Elaz	1
Gaziantep	3364
Hatay	5696
Kahramanmara	3752
Kilis	273
Malatya	2285
Osmaniye	223
Anlurfa	71

To have an idea about the possible impact on air pollution, the first research step was addressed to academic journals and scientific publications: Scopus, PubMed, IEEE Xplore, and Google Scholar were used to identify peer-reviewed articles. Relevant keywords in the abstract “earthquake,” “air pollution,” and “Turkey” were used. Due to the limited availability of scientific data (only two papers were found (Bayram et al. 2023; Mohammad et al. 2023)), a diverse range of sources was considered. They included government reports, media sources, NGO, and international organizations reports. The aim was to offer a more comprehensive analysis, providing a suitable understanding of the topic and helping to formulate informed research questions for your study.

A different search approach was also made by searching only for “earthquake” and “air pollution” in the SCOPUS database, excluding works devoted to volcanic events. The already published papers resulted to 101. By reading all the abstracts, it resulted that only four papers are addressed to treat the problem of air pollution, due to an earthquake. They are reported in Table 2 with a resume of some effects generated during earthquake. Two of these papers also report some solutions for the control or prevention of dust.

**Table 2:** Papers that discuss the problem of air pollution correlated with earthquake

Reference	Effect of earthquake on air pollution	Solution for dust control/prevention
Moslehi et al. (2020)	After the earthquake, people take refuge in their cars. Vehicles represent one of the main factors that increase the concentration of air pollution	-
Yan and Galloway (2017)	Diffusion of radioactive substances and asbestos fibers	Easy access to measuring instruments to check the status of personal air quality in an emergency, with priority to children, the elderly, and the sick
Uchiyama (2013)	Large quantities of dust generated by collapsed buildings and rubble during post-disaster reconstruction	Control dust generation by sprinkling water, enforce the wearing of dust mask, and avoid dusty places, especially for children and elderly
Gotoh et al. (2002)	High concentration of dust during demolition works. The analysis of total suspended particulate concentration, analysis of concrete, mortar, and soil dusts samples, and evolution of alkalinity of concrete dust were performed	-

Concerning the possible air pollution sources, apart from the buildings also mining activities have been considered. Indeed, Turkey possesses several mine sites, with a rich array of minerals, ranking 10th globally in terms of mineral variety and 28th in underground resource production, with 60 different types of minerals (IFLR1000 2023). Among these minerals, raw materials utilized in the construction, cosmetics, and agriculture sectors hold particular importance. Mining exports contribute significantly to Turkey’s gross domestic product. Copper, chrome, coal, marble, and boron are key minerals that hold a prominent position in the market. The country’s extensive and diverse mineral resource base encompasses coal, gold, iron, lead, mercury, silver, and tin, as well as other precious metals and coal.

To evaluate air pollution generated by dust and powders from mining sites, particulate matter (PM) is considered: PM stands out as a significant air pollutant, originating from both human activities and natural phenomena. The morphology and chemical composition of PM are intricately linked to their source (Bontempi et al. 2010). Several PM detection techniques are available in the literature (Borgese et al. 2020).

This multifaceted approach laid the groundwork for comprehending post-earthquake air pollution, facilitating the formulation of research questions that are informed and insightful.

**Fig. 2:**
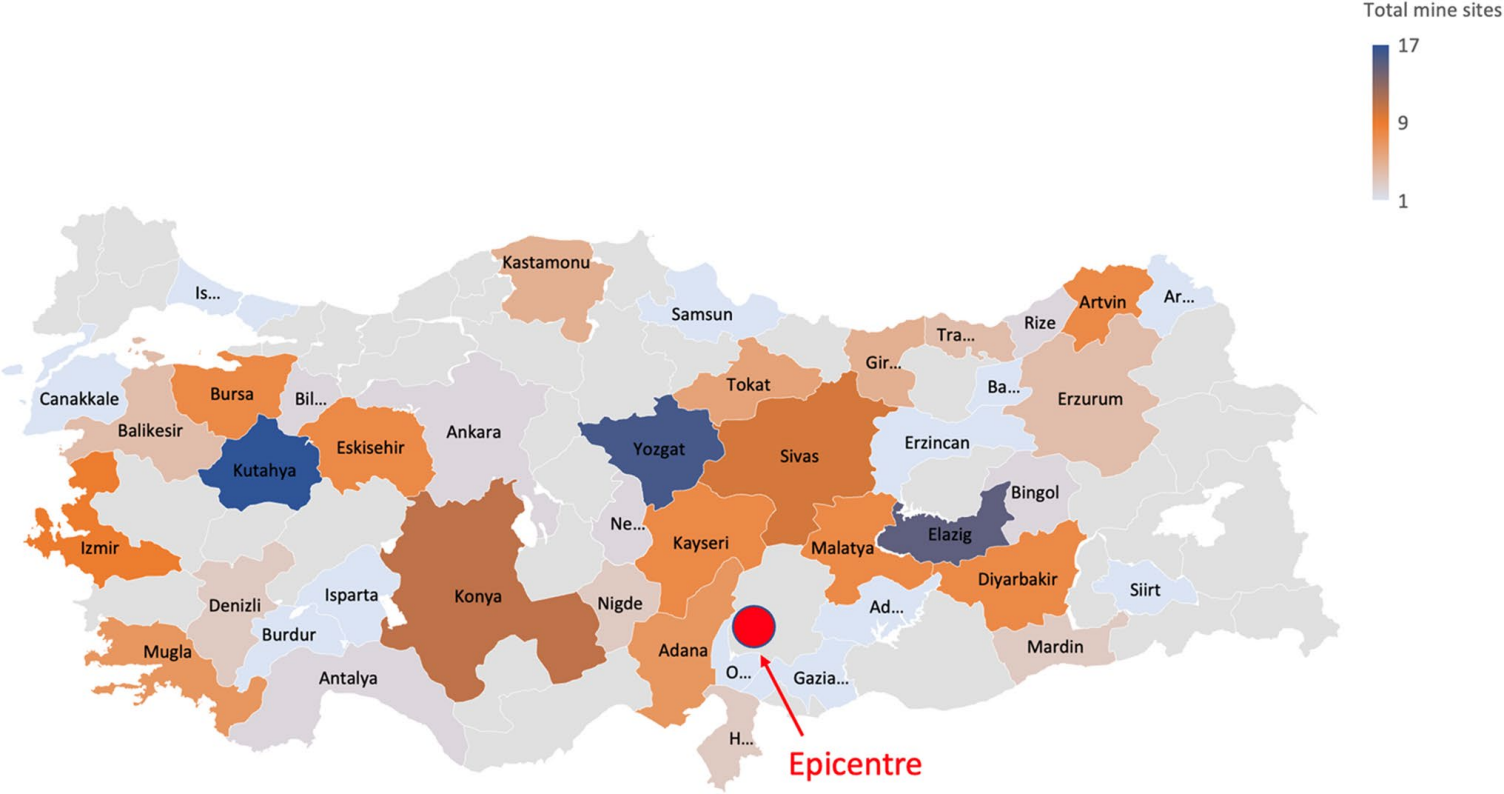
The total number of mining sites in the Turkey regions

Figure 2 shows the total number of mine sites in Turkey (Diggings 2023). In 2022 Turkey extracted 24,520,000 tons of metal ores and 50,730,000 tons of non-metallic ores (Statista 2023).

## Results and discussion

### The earthquake’s impact on the air quality

Scientific literature about the effect of air pollution in Turkey after the 2023 earthquake is very limited. Based on the study design results, only two peer-reviewed papers were found. In particular, one paper reports an evident increase in respiratory visits to the Antakya hospital in Turkey, but they are based on personal communication with a University Hospital researcher (Mohammad et al. 2023). The second work (Bayram et al. 2023) reports general information about the possible air contamination, due to dust and other PM from collapsed buildings. However, in the available existing scientific literature, no information about the possible air pollutants generated by this earthquake is reported.

There are several mechanisms by which seismic activity affects air quality. One mechanism is the release of trace gases, such as SO_2_, due to seismic-triggered degassing (Hsu et al. 2010). Seismic activity can cause the uprising of geogas toward the surface (Sciarra et al. 2017; Walia et al. 2010). Moreover, the earthquake primarily generates air pollution, particularly from destroying buildings and infrastructure.

Additionally, heavy machinery and equipment used after the earthquake can further contribute to air pollution. Pre-operational hydraulic fracturing activities can elevate air pollution, particularly NOx, due to construction work on site (Wilde et al. 2023). The earthquake in Turkey significantly impacted air pollution, as it caused the release of hazardous substances into the air, such as dust and debris from the damaged buildings.

Data from the European air quality analysis of the Copernicus Atmosphere Monitoring Service showed that starting from February 7, there was an increase in PM_2.5_ concentration, which can be generated by debris from the earthquake. Later, between February 12 and 14, as well as after February 22, the high PM_2.5_ concentrations (reaching values higher than 70 μg/m^3^ for several days) were reported (Mohammad et al. 2023). This may be associated with aerosol resuspension, due to dust and powders originating by debris and during demolition activities. Hazardous substances are typically removed from buildings before their demolition in standard practices. However, earthquakes render this practice impossible. In the case of Turkey, it was estimated that about 300,000 buildings either collapsed, required demolition, or sustained moderate damage (Toksabay et al. 2023), originating between 116 and 210 million tons of rubble (UNDP 2023). The dust and debris from the earthquake can lead to respiratory problems and other health issues.

**Fig. 3:**
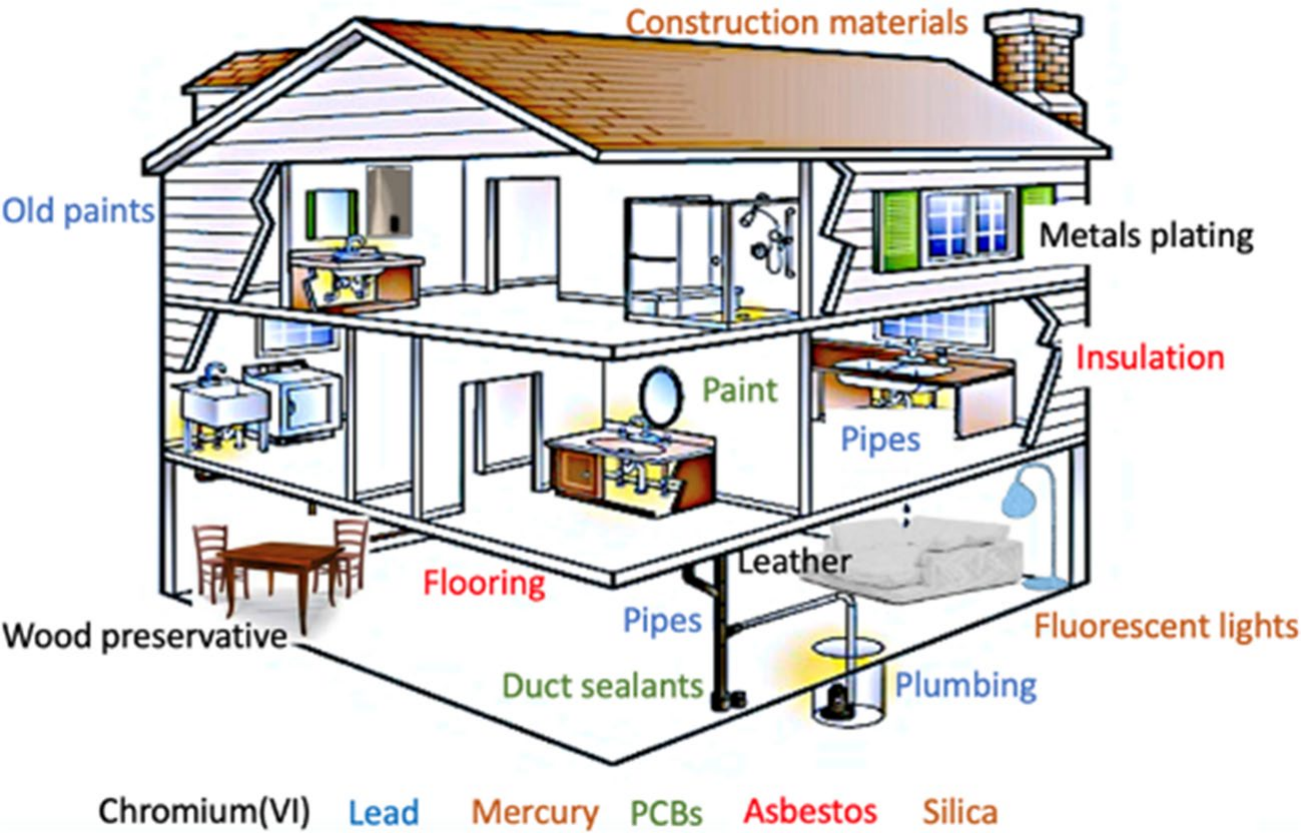
Possible contaminants originating from buildings (chromium, lead, mercury, PCBs—polychlorinated biphenyls, asbestos, and silica), based on the data about the corresponding source materials in Turkey

Apart from the fine powders made of cementitious materials, some pollutants have been dispersed in the air, due to the earthquake. Indeed, hazardous materials exist in different parts of a building, including paint and pipes. Some are prominent examples of these pollutants and the potential areas where they might be found are discussed. They are asbestos, PCBs, silica, and lead. Other pollutants can originate from both wastes and mining sites, like mercury and Cr(VI), i.e., hexavalent chromium. Figure 3 shows the potential contaminants originating from buildings.

They are selected based on the data available for the availability of the corresponding material in Turkey. A recent publication has shown that in Turkey, asbestos was found in outdoor urban and rural environments (Nasirzadeh et al. 2020), before the earthquake.

The indoor asbestos concentration was found to be higher than the standard level, corresponding to 0.1 f/ml, and the outdoor reached values of 2.57 f/ml, mainly due to the exposition of asbestos-containing white soils (Bolan et al. 2023). Indeed, the annual survey of the US Geological about asbestos consumption data reports that a total of 111,123 tons, corresponding to 11,112 tons per year, of asbestos were consumed in Turkey between 1999 and 2009 (Stayner et al. 2013).

These data support some evidence: 6 months after the earthquake, the Guardian (Guardian News and Media 2024) reported some pictures, showing mountains of rubble and asbestos blighting the Turkey landscape. The presence of asbestos was attributed to the extensive use of this material in cement construction.

This can allow us to conclude that the exposition to this pollutant will increase in the next months, due to the applied procedures of building waste management. The estimate of the Chairman of Turkey’s Association of Asbestos Dismantling Experts suggests that at least 3 million people will fall ill in the next years, only due to asbestos exposure. Silica is also a common material used in construction, like concrete, bricks, and gardening suppliers. The prolonged exposure to fine silica particles can cause serious respiratory diseases and cancer.

Recent literature shows that it is an already recognized problem in Turkey, due to erosion caused by winds and streams (Stayner et al. 2013). Earthquakes can only exacerbate the problem.

Other air pollutants may originate from electronic equipment and mining activities. Several studies have shown that polychlorinated biphenyls (PCBs) have toxic effects due to chronic and acute exposures. Some commercial products contain PCB such as capacitors, transformers, and lubricants. Despite their prohibition a long time ago around the world, they can still be found in the environment due to their chemical stability, bio-accumulation properties, and low solubility in water (Reddy et al. 2019). Literature reported the presence of 1080 tons of PCBs in materials and equipment in Turkey in 2013 (Heinzow et al. 2007; Sari et al. 2020). The mining and quarrying industry extracts minerals, oil, gas, and other natural resources from the Earth’s surface, making it a crucial contributor to the global economy. This sector provides vital raw materials necessary for industries such as construction, manufacturing, and power generation.

When extreme events occur, this can be the source of environmental pollution, due to mining activities: an earthquake has the potential to create air pollution issues, due to the damage to mining infrastructure, resulting in the dispersal of dust and other pollutants into the atmosphere. Moreover, the tremors and disturbance of soil and rock during an earthquake can generate dust clouds containing potentially harmful particles (US Geological Survey 2023a). An example concerns mercury deriving from mining sites: Turkey has a large number of mercury reserves, with several old mining sites. Indeed, in 1974, Turkey produced more than 12,000 flasks of mercury, corresponding to about 5% of the world’s output (Arik 2022). In 2020 the export of mercury resulted in a value of $339,000 for Turkey (World Bank 2023), resulting in one of the leading mercury-exporting countries worldwide. Mercury contamination, which can originate from the earthquake, impacting both active and closed mining sites, results in a global issue that may impact not only air but also water bodies. This is due to the widespread dispersion of mercury emissions in the atmosphere, which then settle over various regions, and it is extremely hard to quantify it. In addition, mercury can be also found in fluorescent lamps, in ancient buildings. Also Cr(VI) can be present in certain applications such as pigments, dyes, metal plating, leather, cement, and wood preservatives. It is evident that Cr(VI) contamination can arise from building waste. However, Turkey is well known also for its chromite resources, where there are several open pit mines, with a production of 6900 tons of chromium in 2022. Another mined metal in Turkey is lead. It is an element found naturally in the environment, possessing characteristics of softness and malleability. It was used in bulging applications, for example, for lead-containing products, plumbing and manufacture, and to produce painting. Turkey produced about 75,000 tons of Pb in 2022 by mining, as reported in Table 3.

**Table 3:** Data about materials originating possible pollution sources in Turkey, caused by earthquake event

The potential pollutant source	Amount (tons)	Origin	The time	Reference
Chromite	6900	From mining	2022	US Geological Survey (2023b)
Pb	75,000	From mining	2022	US Geological Survey (2023b)
Metal ores (total)	24,520,000		2022	Statista (2023)
Non-metallic ores (total)	50,730,000		2022	Statista (2023)
PCBs	1080	Electronic materials and equipment	2013	Sari et al. (2020)
Asbestos	11,112 (tons per year)	Cement construction	from 1999 to 2009	Stayner et al. (2013)
Oil refinery throughputs (PAHs)	691 (thousand barrels per day)	Petroleum and refinery activities	2021	Statista (2023)

*PAHs*, polycyclic aromatic hydrocarbons; *PCBs*, polychlorinated biphenyls

Lead can enter the soil and it can persist for long periods, posing a threat to plants, animals, and ultimately human beings. It can accumulate in crops and vegetation, making it a pathway for human exposure.

Upon release in the atmosphere, lead has the potential to traverse considerable distances in the air before ultimately settling on the ground, typically adhering to soil particles. Then people can be exposed to Pb also by breathing air.

### Health effects of air pollution

After the earthquake, an increase in respiratory problems was reported by the Antakya Hospital (Mohammad et al. 2023).

**Fig. 4:**
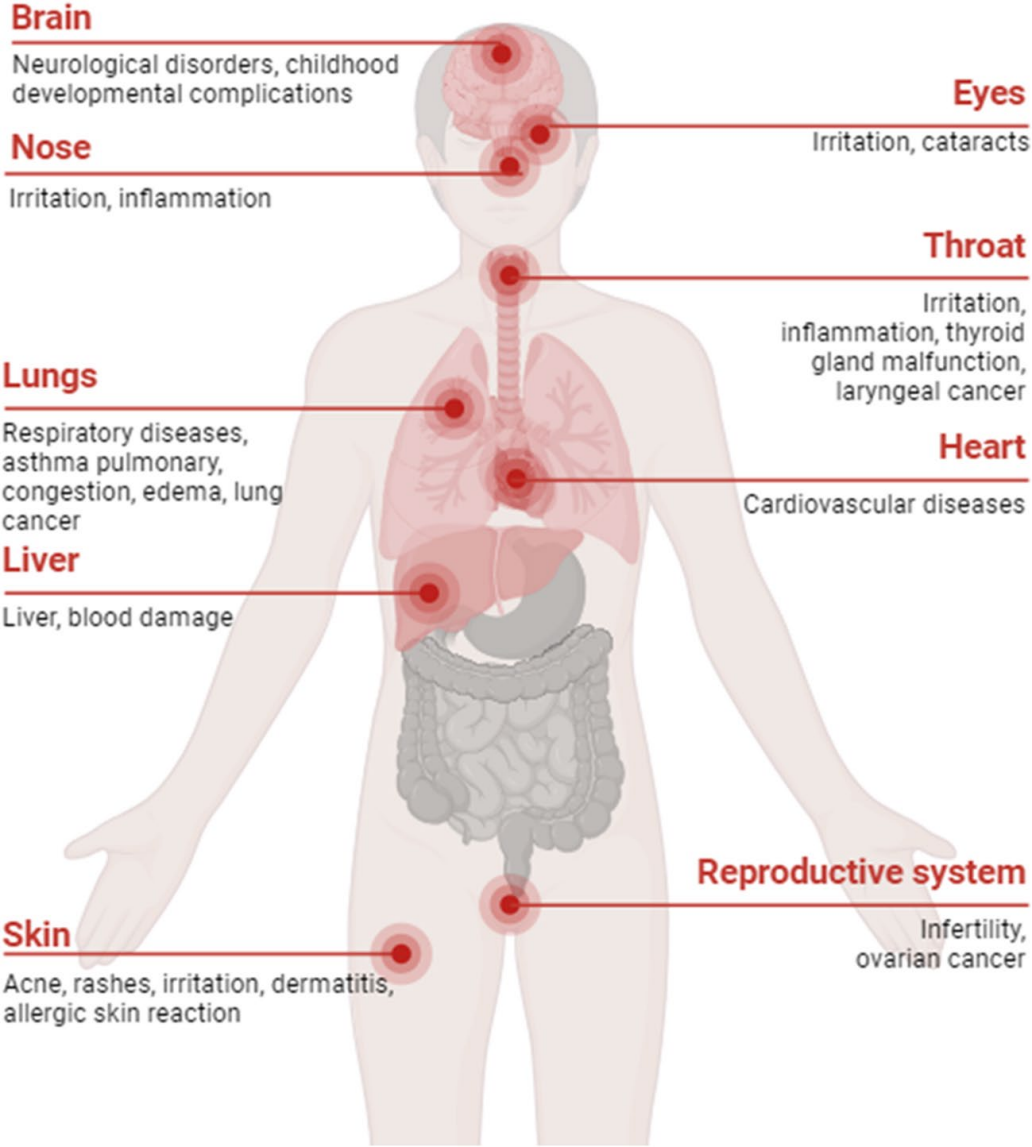
Possible health effects generated by the air pollutants that may be found in the air after the Turkey earthquake. Air pollution is frequently imperceptible until its severe consequences become evident later on. The figure was created with BioRender.com

Moreover, the pollutants generated by the disaster can produce different health effects (see Fig. 4), frequently imperceptible until its severe consequences become evident later on. The harmful nature of these particles is determined by their composition and aerodynamic diameter, with the latter being the primary limiting factor for their ability to penetrate deeper into the respiratory tract.

The small fibers and clumps of asbestos can be released into the ambient air as airborne particles. This exposure is the primary cause of mesothelioma. This cancer forms in the lining of the lungs or abdomen. Other types of cancer caused by asbestos include lung, ovarian, and laryngeal cancer.

Inhaling PCBs can lead to various adverse effects, such as respiratory problems, neurotoxicity, and immunotoxicity (Kraft et al. 2021). PCB can also interfere with the normal functioning of the thyroid gland and have harmful effects on the reproductive system (Kraft et al. 2021). Negative effects can also be found in the skin (such as severe acne and rashes) and the cardiovascular system.

The short-term exposure to PAHs can caused respiratory disease and thrombotic effects in people affected by coronary disease. The long exposure to PAHs can also generate eye and skin irritation, nausea, and cancer. It can also induce cataracts, and liver and kidney damages (Kim et al. 2013).

Exposure to Cr(VI) can lead to various adverse health effects, including asthma, respiratory and eye irritations, liver damage, pulmonary congestion and edema, nasal irritation and damage, and respiratory cancer. In addition, some individuals may develop allergic contact dermatitis, an allergic skin reaction, particularly among workers (US Department of Labor 2023; Iyer et al. 2023).

The harmful effects of airborne Pb on human health include neurological disorders, developmental delays, cognitive impairments, lowered intelligence quotient levels, and increased risk of cardiovascular diseases. Lead exposure can also impact the respiratory system, causing respiratory irritation, reduced lung function, and respiratory conditions such as bronchitis.

The severity of several of these negative effects can depend on the dose and the duration of the exposure. Indeed, some of these effects can be associated with long-term exposure to high levels of pollutants (Yang et al. 2021).

The increased air pollution can lead to respiratory problems and other health issues for the local population, particularly those with pre-existing conditions. To mitigate the earthquake’s impact on air pollution, it is crucial to implement measures to reduce emissions and improve air quality.

The waste storage sites must be placed under secure management, and an inventory must be maintained to track incoming waste. To prevent contamination of the air (and also of the soil and the water), as well as the spread of diseases, it is crucial for Turkey to effectively manage the waste resulting from the earthquake.

### Actions for the alleviation and understanding of air pollution effects

Pollution prevention to obtain risk mitigation is mandatory in the case of an earthquake. It is evident that it is not possible to completely avoid any pollution risks, but actions devoted to their mitigation are imperative. There are no specific studies concerning measures for air pollution mitigation due to disasters, not only for earthquakes but also, for example, for volcano eruptions. Moreover, some actions can be proposed, based on the possible pollutants and their health effects.

The first actions must be devoted to safeguarding the population, due to the worsening of air quality, by making available respiratory protection. Despite that few studies have investigated the efficacy of facemasks, outside of occupational settings, their use should be recommended (McDonald and Horwell 2021). Moreover, some studies reveal that different respiratory protections offer notably distinct levels of defense, depending on the size and the composition of dust particles, the effectiveness of the material in filtering particles, and how well the protection fits the face. Then, more research should be devoted to better studying respiratory protection, depending on the disaster (for example, earthquake or volcano eruption). Suitable devices should be acquired by the political authorities of areas with a high risk of these events.

In order to alleviate environmental risks and the resulting health threats, it is crucial to adopt effective waste management procedures in the aftermath of the catastrophe. This includes the safe removal, storage, and disposal of debris, as well as the identification and remediation of any contaminated sites. Adequate measures should be taken to prevent the spread of pollutants and minimize the long-term impact on the environment. For example, wherever possible, the safe removal of asbestos-containing material should be considered, by using in-situ immobilization techniques.

Various techniques can be employed in specific locations, also in the proximity of mining sites, to mitigate the dispersion of fine particle clouds. The dust control method, known as soaking, involves saturating the dust-generating material with large amounts of water to prevent the formation of airborne dust particles. Equipment such as fire hoses, industrial sprinklers, and water trucks are commonly used for this purpose. While soaking reduces dust generation during operations, it has some limitations. A more efficient and water-saving approach to dust prevention is the use of misting cannons. Unlike soaking, misting cannons offer several advantages. They create a fine mist that effectively seals the material’s surface without excessive saturation. The small particle size of the mist minimizes standing water and on-site run-off. Misting cannons act as stationary dust prevention devices, ensuring effective dust control while conserving water. Foam can also be employed as an effective dust prevention method. It is applied to dust-generating areas, covering the materials, and preventing dust dispersion by natural forces. Foam application involves mixing additives with water and delivering the foam through spray bars. It provides an additional layer of protection and control against dust. Dust preventative surfactants offer another option for dust prevention. These chemicals, when mixed with water, bind to the surface of dust-generating materials, creating a protective barrier. The reaction between the surfactant and water forms a crust on the material’s surface, offering resistance against mild mechanical disruption and wind effects. By implementing these diverse dust control methods, such as misting cannons, foam application, and dust preventative surfactants, Turkey could effectively manage dust generation, reduce water consumption, and mitigate the environmental and health impacts associated with airborne dust particles, generated by earthquake waste management. It will be also fundamental to strict control of materials from more polluted locations, for example, in the proximity of mines and refining centers and devoted to massive transfer for reconstruction.

Due to the relevant implications of the disaster, it is fundamental to check the long-term impacts on the health of affected populations. Indeed, as already reported, the severity of several of these negative effects can depend on the dose and the duration of the exposure. Regular health monitoring and surveillance of people exposed to hazards can help in the early detection and treatment of health issues.

Implementing public health interventions, which include the dissemination of information on protective measures for individuals who may have been exposed, holds considerable benefits. Beyond the immediate health implications, these initiatives contribute to the broader social fabric by fostering a sense of collective responsibility and awareness. By empowering individuals with knowledge on safeguarding their well-being, such interventions not only address health concerns but also enhance community resilience and promote a culture of mutual support. In times of uncertainty or crisis, this shared understanding and proactive approach contribute to a more cohesive and resilient society.

Enhancing the understanding and anticipation of health effects requires knowledge of the population’s exposure levels. Severe air quality episodes pose a significant global threat to human health, but their dynamic nature makes monitoring exceptionally challenging. Dedicated monitoring platforms, such as a network of low-cost sensors (Crawford et al. 2021), can capture these episodes. The utilization of low-cost monitoring systems holds the potential to provide detailed estimates of human exposure to certain pollutants after an earthquake, even in cases where support from pre-existing air quality monitoring systems is lacking. Attention must be paid by institutional and political authorities to support the understanding of these phenomena.

Enforcing policy and regulatory measures to safeguard environmental health and safety amid and following disasters is also crucial. This can be based on the availability of data about air pollution and health issues.

Finally, suitable information and data sharing is an essential step to better manage the situation. It is noteworthy that the US Environmental Protection Agency (EPA) encountered controversy regarding its management of air quality information in New York City around Ground Zero after the attacks. Moreover, the lack of information about the air quality must be avoided by institutions.

## Conclusions and perspectives

During an earthquake, buildings may release hazardous substances into the environment, such as asbestos, lead, and other toxic materials. These pollutants can contaminate the air, affecting ecosystems and public health. Furthermore, the improper disposal of building waste, such as illegal dumping or inadequate storage facilities, can exacerbate the pollution issue. It can lead to the leaching of harmful substances into the soil and groundwater, as well as the release of dust and pollutants into the air, contributing to air pollution.

Some measures are proposed to mitigate the effects of extreme events on air pollution. Moreover, this work shows that a comprehensive understanding of the risks and consequences of earthquakes on air pollution necessitates crucial scientific activities and research. Then, also the utilization of monitoring systems with the potential to provide detailed estimates of human exposure is suggested. Other actions involve regular people health monitoring and surveillance, dissemination of information, enforcement policy and regulatory measures, and information sharing.

Feasible measures should be contemplated to empower public authorities to effectively respond to emergencies, especially in situations of disasters that result in the release of airborne dust particles.

This study has some limitations:

- Due to the availability of few works on this matter, some of the bibliographies refer to different natural disaster contexts.
- It is noteworthy that a more in-depth investigation would be necessary to assess the long-term health repercussions of such occurrences. Then several years will be necessary to evaluate the paper’s impact.
- Detailed air pollution data during and after the earthquake are not available.
- The information about the impact of certain human activities, such as mining, on air pollution during extreme events remains incomplete.

## Data Availability

Data will be made available on reasonable request.
